# Vegetable disease detection using an improved YOLOv8 algorithm in the greenhouse plant environment

**DOI:** 10.1038/s41598-024-54540-9

**Published:** 2024-02-21

**Authors:** Xuewei Wang, Jun Liu

**Affiliations:** Shandong Provincial University Laboratory for Protected Horticulture, Weifang University of Science and Technology, Weifang, China

**Keywords:** Greenhouse plant environment, Vegetable diseases, YOLOv8n, Object detection, Attention mechanism, Biological techniques, Plant sciences

## Abstract

This study introduces YOLOv8n-vegetable, a model designed to address challenges related to imprecise detection of vegetable diseases in greenhouse plant environment using existing network models. The model incorporates several improvements and optimizations to enhance its effectiveness. Firstly, a novel C2fGhost module replaces partial C2f. with GhostConv based on Ghost lightweight convolution, reducing the model’s parameters and improving detection performance. Second, the Occlusion Perception Attention Module (OAM) is integrated into the Neck section to better preserve feature information after fusion, enhancing vegetable disease detection in greenhouse settings. To address challenges associated with detecting small-sized objects and the depletion of semantic knowledge due to varying scales, an additional layer for detecting small-sized objects is included. This layer improves the amalgamation of extensive and basic semantic knowledge, thereby enhancing overall detection accuracy. Finally, the HIoU boundary loss function is introduced, leading to improved convergence speed and regression accuracy. These improvement strategies were validated through experiments using a self-built vegetable disease detection dataset in a greenhouse environment. Multiple experimental comparisons have demonstrated the model's effectiveness, achieving the objectives of improving detection speed while maintaining accuracy and real-time detection capability. According to experimental findings, the enhanced model exhibited a 6.46% rise in mean average precision (mAP) over the original model on the self-built vegetable disease detection dataset under greenhouse conditions. Additionally, the parameter quantity and model size decreased by 0.16G and 0.21 MB, respectively. The proposed model demonstrates significant advancements over the original algorithm and exhibits strong competitiveness when compared with other advanced object detection models. The lightweight and fast detection of vegetable diseases offered by the proposed model presents promising applications in vegetable disease detection tasks.

## Introduction

The detection of plant disease objects plays an essential role within the realm of plant protection, as it directly influences the effectiveness of disease prevention and control. Detecting plant disease objects poses unique challenges compared to general object detection tasks. Environmental factors such as scale, angle, and lighting, as well as issues such as background interference, noise interference, low imaging resolution, and significant variations in target morphology and distribution, require the use of advanced models^1–3^. Unlike conventional techniques that depend on manual feature design and extraction for detection, the rapid advancement of deep neural networks offers a more promising approach. Intelligent algorithms capable of self-sensing, adaptivity, self-organization, and self-coding represent the future research direction in this field.

Object detection algorithms fall into two main categories: vision transformer methods based on self-attention mechanism neural network models and CNN-based methods. Vision transformer methods, such as DETR^4^, deformable DETR^5^, and DAB-DETR^6^, utilize the self-attention mechanism to capture global dependencies within images, leading to dense object detection predictions. However, a notable drawback is the high computational complexity associated with these algorithms, demanding substantial computational resources and time. Consequently, researchers in this domain focus on expediting convergence and reducing weight.

In the realm of CNN-based methods for object detection, a fundamental classification involves two-stage and one-stage detection algorithms. Two-stage detection algorithms, typified by the RCNN series and its derivatives, prioritize detection accuracy by sacrificing speed and increasing model complexity^7,8^. In addition to these, noteworthy alternatives such as Detic^9^, DiffusionDet^10^, and EfficientDet^11^ have emerged.

On the contrary, the second category comprises one-stage detection algorithms, exemplified by the YOLO series and its derivatives. These algorithms aim for a balance between precision and speed, emphasizing an optimal equilibrium. Single-stage object detection algorithms, like the YOLO series, contribute significantly to research in object detection algorithms. Recent iterations such as YOLOX^12^, YOLOV6^13^, YOLOV7^14^ and YOLOV8^15^ have showcased superior performance across various aspects of object detection. This includes advancements in backbone extraction networks, fusion modules, data augmentation processing, detection head modules, anchor frame design, loss function optimization, training strategies, and deployment methods.

However, given the intricate nature of the environment for detecting plant diseases, including drastic changes in object scale, strong background interference such as light, and low resolution, many algorithms still face limitations within the domain of plant disease detection despite the high cost of manually annotated training data. Hence, there exists potential for enhancing detection performance^16^.

The importance of accurate and timely plant disease detection cannot be overstated^17^. Early disease identification is essential for implementing effective control measures, thereby minimizing crop losses and reducing the need for chemical interventions^18^. Over the years, researchers have explored a range of methodologies and technologies to enhance the precision, speed, and scalability of plant disease diagnosis^19,20^.

Researchers have focused their attention on different crop varieties, including staple cereals, fruits, vegetables, and cash crops, tailoring their approaches to the specific requirements of each plant type. Moreover, they have investigated various disease types, from bacterial and fungal infections to viral and nematode-induced diseases^21^. This diverse array of studies has enriched our understanding of plant-pathogen interactions and paved the way for the development of targeted disease management strategies.

In the domain of vegetable disease recognition, Ullah et al^22^. introduced a fusion approach that combines two pre-trained models to improve the performance of detecting diseases in the context of small and medium-sized cases. They achieved an impressive precision of 99.92% on a simple background tomato disease dataset. On the other hand, Albahli et al^23^. proposed the CornerNet algorithm based on DenseNet-77, which achieved a maximum accuracy of 99.98%. However, this algorithm has a complex network design, and due to the abundance of parameters, it is impractical to deploy it on portable gadgets. Consequently, these methods may not be suitable for the detection of vegetable disease in greenhouse environments where mobile deployment is desired.

Saleem et al^24^. developed a vegetable dataset called NZDLPlantDisease-v2 and reached a mean average precision (mAP) value of 91.33% using the Faster RCNN Inception ResNet-v2 model. Furthermore, Zhao et al^25^. collected a dataset consisting of images depicting tomato diseases and cucumber diseases under challenging background conditions. They used transfer learning techniques to build a DTL-SE-ResNet50 model for identification purposes of vegetable diseases. This approach leverages the knowledge from pre-trained models and fine-tunes them for detecting vegetable diseases. The studies mentioned above contribute to the advancement of vegetable disease detection by providing specialized datasets and proposing effective models for the accurate detection of vegetable diseases.

The application of attention mechanisms has brought significant improvements to the precision of vegetable disease detection. Qi et al^26^. proposed the use of the squeeze and excitation module to improve YOLOv5 detection performance on small disease objects affected by tomato virus disease. Jing et al^27^. introduced the CBAM attention mechanism^28^. to improve the tomato disease detection method based on YOLOv5. However, this mechanism operates sequentially on channel and spatial attention, neglecting the interrelation between channel and spatial dimensions, which may result in the potential loss of information across multiple dimensions. To address this limitation, Li et al^29^. employed the coordinate attention (CA) mechanism to develop the MTC-YOLOv5n model. This model achieved an mAP of 84.9% on a dataset specifically constructed for cucumber diseases. The CA mechanism allows for better integration of spatial and channel information, enhancing detection performance. Sun et al^30^. constructed a dataset consisting of Chinese feature description statements corresponding to 10 disease images of two vegetables. They introduced the Veg DenseCap model, which employed the Convolutional Block Attention Module (CBAM) to address the issue of sample imbalance and achieved a mAP of 88.0%.

In greenhouse planting environments, various types of diseases affect different vegetables, and the occurrence time, types, and symptoms of the diseases can vary even within a specific type of vegetable^31^. Although several intelligent methods and tools have been developed for the detection of vegetable diseases^32^, achieving accurate detection results in greenhouse environments remains a formidable task due to the intricate nature of the diseases.

Based on previous research experiments and theoretical considerations, we aim to improve the efficiency of object detection algorithms specifically designed for identifying vegetable diseases. To achieve this, we built upon the foundation provided by the YOLO series of one-stage detection algorithms. Our developed vegetable disease detection model, known as YOLOv8n-vegetable, aims to tackle the prevalent problems of reduced precision and overly large model dimensions encountered in traditional network detection methods for vegetable diseases. To improve the overall efficacy of the proposed model, we have incorporated ideas from several algorithms, including YOLOV5, YOLOV6, YOLOV7 and YOLOV8. The integration of these ideas is specifically targeted at enhancing the global context information extraction proficiency. Additionally, we introduced the concept of the occlusion perception attention module, which further improves the detection accuracy. Within our feature fusion module, our focus has been on strengthening attention toward occluded objects while effectively suppressing background features. By doing so, we aim to improve the capability of the model to precisely detect vegetable diseases, even when objects are partially obscured. The main contributions of our research are succinctly outlined in the following manner.

1. The introduction of the Ghostconv module and the newly designed C2fGhost module has resulted in a lightweight network, significantly diminishing the parameter quantity while enhancing the detection performance.
2. Through the incorporation of the occlusion perception attention module into the feature fusion network (Neck), the network can place a greater emphasis on the attributes of occluded disease objects. This strengthens the capability of the model to extract and merge features, as well as increasing its interest in occluded objects.
3. The inclusion of a small-sized object detection layer at the 160 × 160 scale elevates the combination of deep and shallow semantic information, leading to improved accuracy in detecting small-sized objects.
4. Utilization of the HIoU empowers the network to dynamically fine-tune the contribution of individual components within the loss function during various stages. This effectively improves the boundary-box regression performance of the model.

## Materials and methods

### Materials

#### Self-built dataset

**Figure 1:**
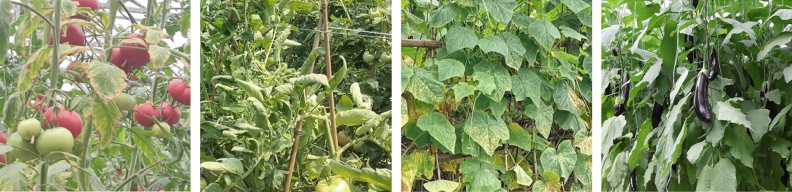
Collected images.

This study used video collection equipment integrated into the online detection system of vegetable diseases developed by the research group. The purpose was to collect disease samples and create a dataset specific to the detection of vegetable diseases within a greenhouse planting environment. In practical planting scenarios, vegetable diseases can manifest in various parts of different plants. Therefore, the positioning of the monitoring camera equipment significantly influences the results of disease detection. To address this, six sampling cameras were installed in different locations within vegetable greenhouses. This approach ensured the collection of videos that capture vegetable diseases from diverse perspectives. Figure 1 illustrates the sample of images collected. Given the relatively low rate of occurrence of vegetable diseases in a short period, it was necessary to optimize the efficiency of sample collection. As a result, vegetable disease samples were continuously collected at different times, resulting in a collection of 800 video sequences. From these sequences, a total of 40,000 keyframe images were extracted.

Based on the provided figure, it is evident that the proportion of vegetable disease objects within the monitoring image is relatively small, typically less than 10%. Consequently, direct detection of vegetable disease based on the original image proves to be challenging. To emphasize disease characteristics, the construction of the dataset involved grouping and cropping of the original images according to different types of disease and infection locations. The cropped area is centered around the disease infection region, with efforts made to include only the complete disease infection area as much as possible. The dimensions of the cropped images are approximately 640 × 640 pixels. Additionally, a manual screening was conducted to eliminate low-quality images, ensuring a balanced sample size between different types of disease. This process yielded 28,000 images. From the images obtained, a subset containing various types of disease was selected, and the disease objects in the cropped images were annotated following the construction standards of the PASCAL VOC dataset. In each image, there are 1 or more annotation boxes for the area infected with diseases.

After evaluating the quantity and quality of annotations in each category, it was determined that the number of annotations about sudden tomato fall, tomato vertical blight, tomato blight, cucumber black rot, cucumber soft rot, cucumber sudden cucumber fall, eggplant black rot, eggplant soft rot, and the associated targets of these eight disease categories was significantly hindered by plant obstruction in certain situations. Therefore, comprehensively considering these factors, the decision was made to remove these eight categories.

After manual confirmation, the compiled dataset included 20 diseases affecting three different vegetables, as detailed in Table 1. To facilitate training, testing, and validation, the dataset was divided into sets using an 8:1:1 ratio.

**Table 1:** Number of samples collected for different types of vegetable and disease categories.

No	Vegetable type	Disease category	Number of images	Annotation box quantity
A1	Tomato	Healthy	1000	4654
A2	Tomato	Early blight	1000	3280
A3	Tomato	Late blight	1000	5026
A4	Tomato	Gray mold	1000	4154
A5	Tomato	Leaf mildew	1000	3441
A6	Tomato	Leaf spot	1000	3135
A7	Tomato	Ulcer disease	1000	3899
A8	Tomato	Anthracnose	1000	3937
A9	Tomato	Leaf curl	1000	4286
A10	Tomato	Viral disease	1000	3075
B1	Cucumber	Healthy	1000	4593
B2	Cucumber	Powdery mildew	1000	3094
B3	Cucumber	Downy mildew	1000	4129
B4	Cucumber	Brown spot	1000	5063
B5	Cucumber	Anthracnose	1000	3410
B6	Cucumber	Viral disease	1000	4665
C1	Eggplant	Healthy	1000	3439
C2	Eggplant	Verticillium wilt	1000	4794
C3	Eggplant	Brown spot	1000	4017
C4	Eggplant	Viral disease	1000	3411

#### Data enhancement

**Figure 2:**
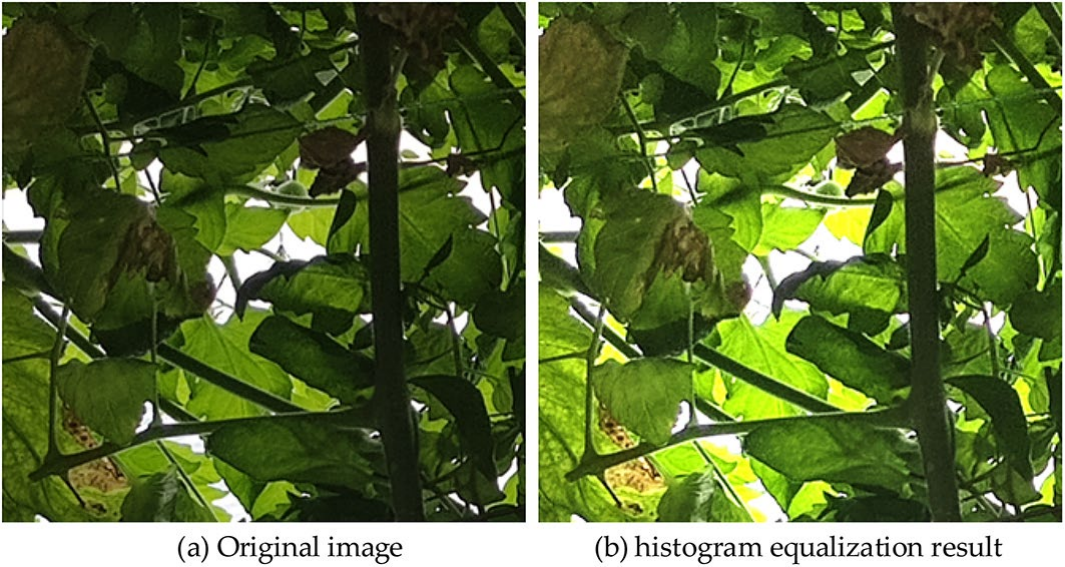
Preprocessing results of low-brightness images enhanced by histogram equalization.

Under meteorological circumstances like cloudy and rainy days, along with environments with overlapping and obstructed plants, the limited light intensity poses challenges in obtaining clear monitoring images. Insufficient detail in the object features of vegetable diseases leads to low accuracy in their detection, as depicted in Fig. 2a. To address this issue, this study employs a fast and effective histogram equalization algorithm for enhancing and preprocessing the input images captured under low illumination, as illustrated in Fig. 2b. The application of this technique significantly improves image clarity, thereby enhancing the capabilities for detecting vegetable disease objects more effectively.

**Figure 3:**
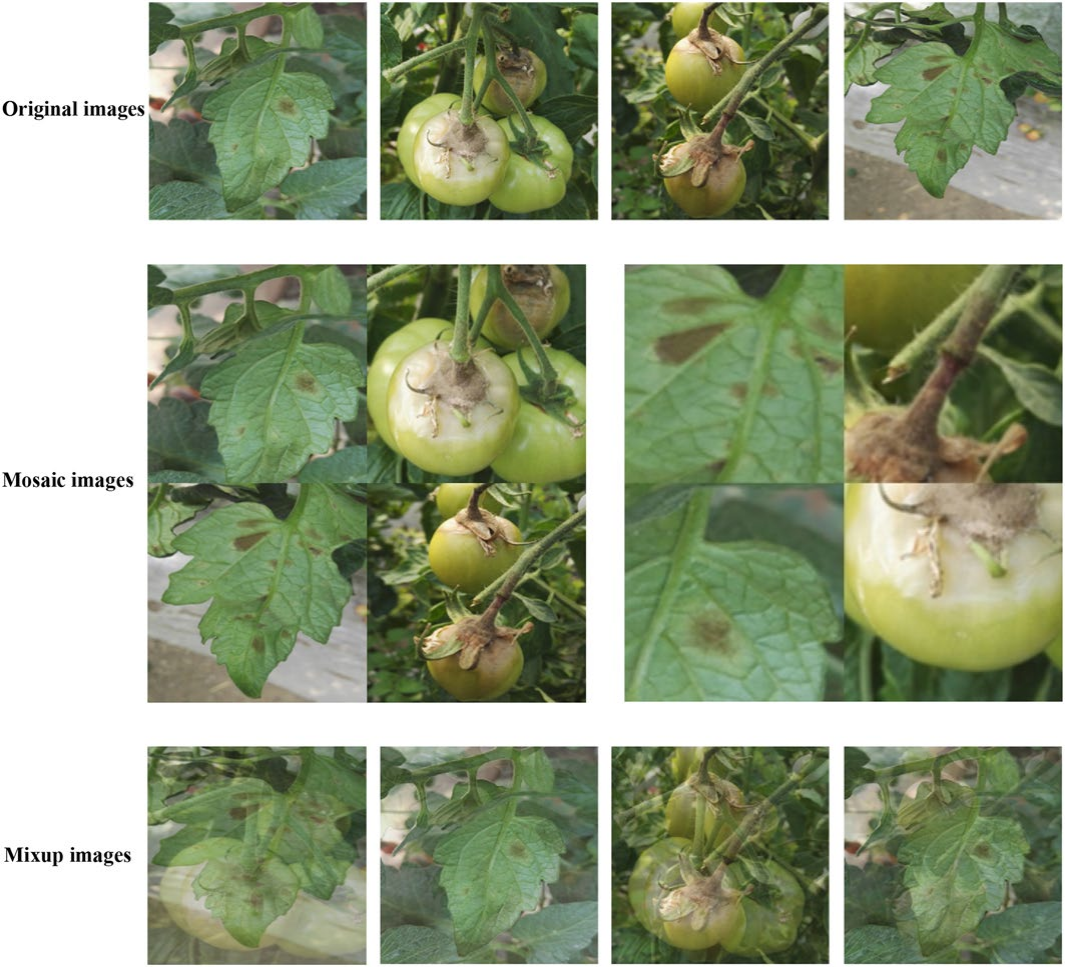
Processing Results of Mosaic and Mixup Data Enhancement Methods.

Furthermore, to enrich the training dataset, we conducted the mosaic and mixup augmentation processes during training. The Mosaic method involves randomly selecting four images from the database, applying random scaling, cropping, and layout operations to each image, and subsequently concatenating them into a new training data image, as illustrated in Row 2 of Fig. 3. On the other hand, the Mixup method randomly selects two images from the training samples and creates a new image through a simple weighted sum operation. The sample labels are also weighted accordingly during this process. This fusion technique helps expand the training data, as illustrated in the third row of Fig. 3.

### YOLOv8n

**Figure 4:**
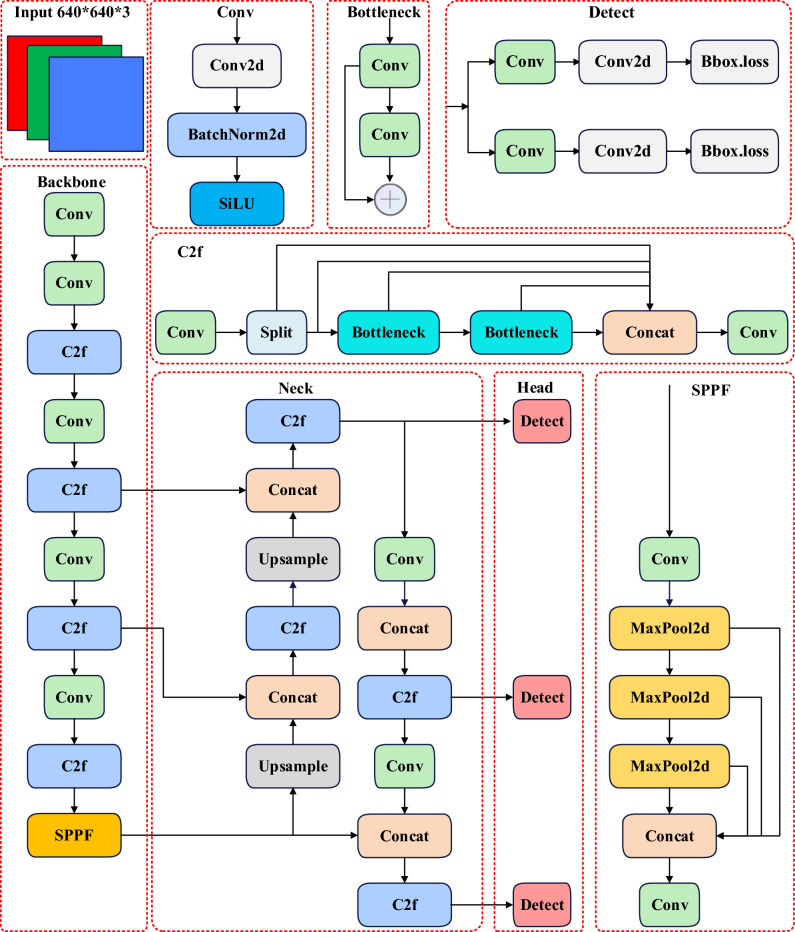
Model structure of YOLOv8n.

Glenn Jocher proposed YOLOv8 as an improvement to YOLOv5. This new model replaced the C3 module (which has a CSP bottleneck with three convolutions) with a more efficient C2f. module (a CSP bottleneck with two convolutions) and adjusted the number of channels. The Head section was also modified to separate classification and detection using the decoupling head technique. Furthermore, the loss function utilized positive and negative matching of samples instead of IOU matching. The YOLOv8 network structure is streamlined, resulting in faster detection speeds and higher detection accuracy. To balance model size and detection accuracy, this study optimized the YOLOv8n version, which has a smaller volume and high accuracy, as shown in Fig. 4.

### YOLOv8n-vegetable

**Figure 5:**
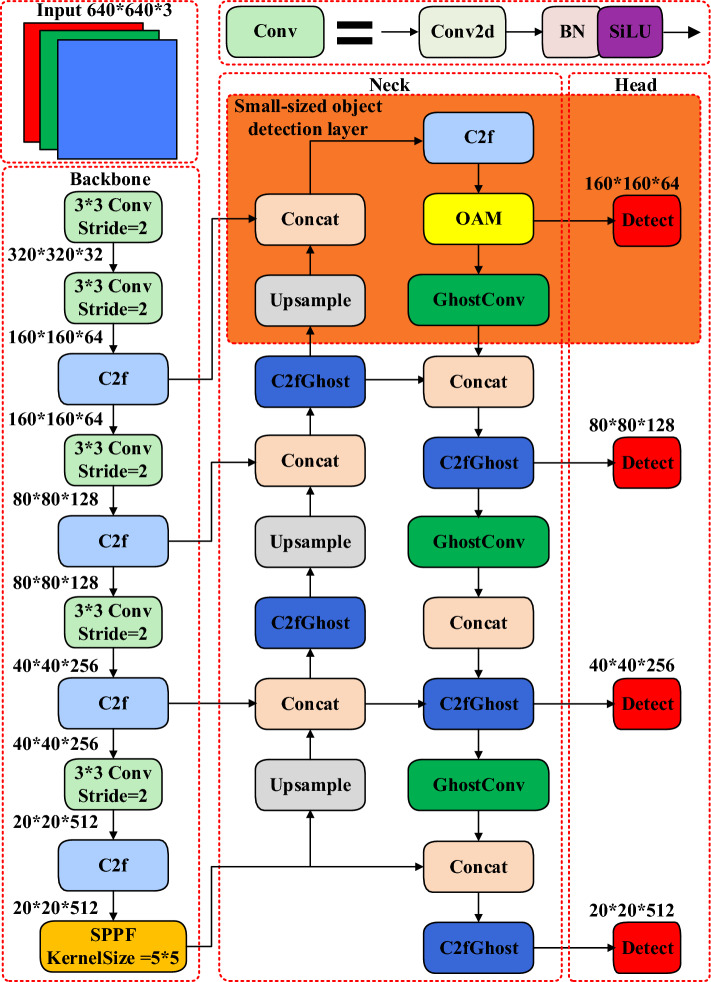
Model structure of YOLOv8n-vegetable.

To tackle issues associated with inaccurate detection of vegetable diseases in conventional networks, excessive model parameters, and large model sizes, this study introduces a novel vegetable disease detection model termed YOLOv8n-vegetable, illustrated in Fig. 5. The model integrates four key enhancements. Firstly, to address the challenge of large model size, GhostConv from the GhostNet network and a newly designed module, denoted as C2fGhost, are introduced to reduce the count of model parameters. Secondly, to enhance the detection of occluded disease objects, an Occlusion Perception Attention Module (OAM) is integrated into the model. Additionally, to cater to the prevalent occurrence of distant objects in the vegetable disease image dataset, often comprising numerous small-sized objects, a specialized layer for small-sized object detection is introduced. Lastly, for optimizing the model's performance in bounding box regression, an HIoU loss function is introduced.

#### GhostNet

**Figure 6:**
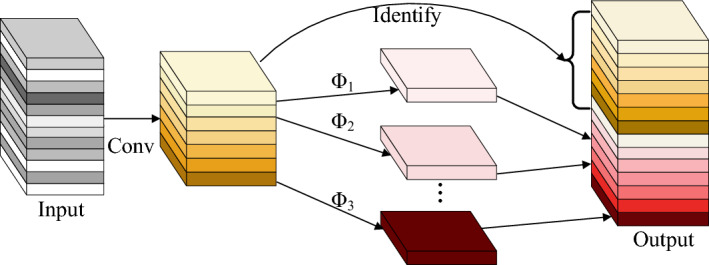
GhostNet^33^.

GhostNet, developed by Huawei Noah Ark Laboratory in 2020^33^, is a lightweight network, aiming to compress the network and streamline the model while ensuring a certain level of model accuracy. As shown in Fig. 6, the GhostNet model preserves channel dimensions while reducing the computational and parameter load of the network. It initially uses a limited amount of ordinary convolution for feature extraction. Following this, linear transformation operations are carried out on the feature map, proving more computationally efficient than regular convolutions. The final feature map is then generated through the Concat operation.

**Figure 7:**
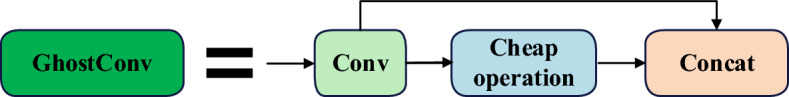
Structure of Ghostconv.

In Fig. 7, Ghostconv emerges as a convolutional module within the GhostNet network, offering a viable substitute for conventional convolutions. The "Cheap operation" embedded therein constitutes an economical linear operation. Diverging from traditional convolutions, Ghostconv orchestrates the parallel execution of feature extraction and cost-effective linear operations, followed by the concatenation of two sets of resultant feature maps. This orchestration serves to further abate computational burdens. Consequently, Ghostconv is purposefully architected as a phased convolutional computing module, yielding an abundance of feature maps through the parallel execution of feature extraction and cost-effective linear operations, thereby exemplifying its computational efficiency. The process begins with GhostConv generating half of the feature map using a convolution with half the size of the original convolution. It then proceeds to pass through a 5 × 5 convolutional kernel and a cost-effective cheap operation with a stride size of 1 to acquire the remaining half. Finally, the two feature maps are concatenated using the Concat operation, resulting in a complete feature map.

**Figure 8:**
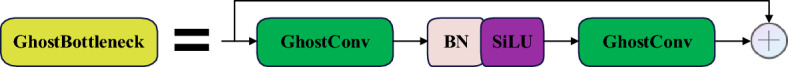
Structure of GhostBottelneck.

Figure 8 illustrates the GhostBottleneck operation. Initially, the quantity of channels is augmented by leveraging the initial GhostConv layer as an extension layer. Subsequently, regularization and Sigmoid Linear Unit (SiLU) are applied to the feature map. Next, the channel count in the output feature map is reduced to match input channels using the second GhostConv layer. Finally, the feature map obtained in the previous stage is added to the residual edge for feature fusion.

**Figure 9:**
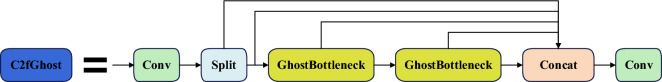
Structure of C2fGhost.

Figure 9 illustrates the newly designed C2fGhost module, which replaces all Bottleneck components in the original network’s C2f. module with GhostBottleneck units. This structure integrates a cross-stage feature fusion strategy and truncated gradient flow technology to enhance the diversity of learned features across different network layers, reducing the impact of redundant gradient information and improving learning capacity. The incorporation of GhostConv and C2fGhost modules effectively diminishes the reliance on numerous 3 × 3 ordinary convolutions in the original structure. Consequently, it significantly compresses the network model's size, decreases the parameter count and computational load, and facilitates deployment on mobile devices. This simplifies the implementation of edge computing for vegetable disease detection.

#### Occlusion perception attention module (OAM)

The attention mechanism plays a crucial role in capturing focal points within the entire image, proving advantageous for extracting small-scale occluded features related to vegetable diseases. However, it is essential to note that utilizing the attention mechanism also entails the drawback of increased computational workload, leading to higher computational costs.

To improve the capability to extract features related to vegetable diseases within the presence of occlusions, a lighter occlusion perception attention mechanism was developed, drawing inspiration from SE (Squeeze and Excitation)^34^, GAM (Global Attention Mechanism)^35^, and Biformer^36^. This design aims to optimize the efficiency of attention mechanisms and improve the network’s performance in occluded scenarios.

**Figure 10:**
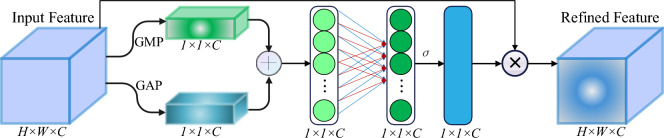
Occlusion Perception Attention Module (OAM).

The Occlusion Perception Attention Module (OAM), illustrated in Fig. 10, improves crucial features related to vegetable diseases by adjusting the weight coefficients for each channel to diminish background information. It employs two pooling operations: Global Average Pooling (GAP) for smoothing out details and preserving the overall distribution, and Global Maximum Pooling (GMP) for capturing local extremum values, especially for small-scale and occluded disease features. By integrating both pooling operations, the mechanism comprehensively considers feature information at various scales, boosting the perception and representation capacity of small-scale occluded disease features. This approach alleviates potential information loss from a single pooling operation. Subsequently, the output undergoes a one-dimensional convolution layer, and the sigmoid activation function is utilized to multiply the matrix with the original feature map, resulting in the ultimate output. In summary, the OAM combines global average pooling, global maximum pooling, one-dimensional convolution, and the sigmoid activation function to effectively capture and enhance critical vegetable disease features while addressing occlusion challenges. The definition of Occlusion Perception Attention is as follows:

1 $$Atten(F)= \sigma \left(\varphi \left(AvgPool\left(F\right)+MaxPool\left(F\right)\right)\right)\cdot F$$

In the above-mentioned formula, F denotes the input feature, φ (∙) denotes a one-dimensional convolutional function, and σ refers to the sigmoid activation function.

#### Small-sized object detection layer

Challenges arise in detecting small-sized objects due to the limited feature information obtained from deep feature maps, which is influenced by both the small size of these samples and the extensive downsampling factor employed by YOLOv8n. Consequently, the original YOLOv8n model exhibits limited detection capability for small-sized objects. In the original model, the dimension of the input image is set at 640 × 640, and the minimum detection scale is 80 × 80. This implies that each grid has a receptive field of 8 × 8. When the vertical and horizontal measurements of an object in the original image are less than 8 pixels, the original network struggles to effectively recognize and extract the object’s feature information within that specific grid.

Therefore, this study incorporates a layer specifically designed for detecting small-sized objects to address these limitations. This layer consists of a 160 × 160 scale small-sized detection layer. It includes supplementary fusion feature layers and additional detection heads to enhance the semantic information and feature expression capability specifically for small-sized objects. The process begins by stacking the 80 × 80 scale feature layer acquired from the fifth layer of the backbone with the upward upsampling feature layer in the Neck section. This combination is subjected to C2 and upsampling operations to generate a deep semantic feature layer that contains essential feature information related to small-sized objects. Next, the shallow position feature layer from the third layer of the backbone is stacked along with the previously obtained deep semantic feature layer. This stacking process improves the expression capability of the resulting 160 × 160 scale fusion feature layer, enabling it to capture both small-sized object semantic features and position information effectively. Finally, this fusion feature layer is passed through an additional decoupling head in the head section of the network, facilitated by C2f. This step further refines the procedure for detecting small-sized objects.

The inclusion of the Head section enables the continuous transmission of feature information associated with small-sized objects along the downsampling path to the other three scale feature layers. This process occurs through the Head structure, thereby enhancing feature fusion and improving the precision of detecting small-sized objects. Moreover, the introduction of additional decoupling heads expands the detection range for vegetable diseases. By incorporating these decoupling heads, the network becomes capable of detecting a wider range of diseases affecting vegetables. Overall, these enhancements result in improved detection accuracy and an expanded detection range, enabling the network to more accurately detect small-scale targets related to vegetable diseases. By incorporating this small-sized object detection layer, the capacity of the model to detect and recognize small-sized objects is significantly enhanced.

#### HIoU loss function

Within the dataset, the presence of subpar instances is unavoidable. Geometric parameters like distance and aspect proportion tend to impose harsh penalties on these low-quality examples, which can negatively impact the model’s generalization performance. To address this issue, an effective loss function must alleviate the penalty for geometric measurements while anticipating substantial overlap between the candidate box and the target box.

Therefore, this study incorporates the concept from Wise loU^37^, which utilizes dynamic methods to calculate IoU losses in category prediction losses. Building upon this idea, a new IoU loss function named HIoU is introduced to optimize the model’s convergence ability and achieve enhanced bounding box prediction regression performance.

Among them, Wise loU addresses the potential bias problem of traditional IoU evaluation and constructs a loss function that employs attention for bounding box refinement. It achieves this by weighting IoU based on the area between the forecasted box and the actual reference box. Let’s assume the predicted box is denoted as B = [x, y, w, h], and the ground truth box is denoted as ${B}_{gt}=\left[{x}_{gt},{y}_{gt},{w}_{gt},{h}_{gt}\right]$. Additionally, let ${W}_{g}$ and ${H}_{g}$ depict the dimensions of the smallest enclosing rectangle between the projected box and the actual reference box, and IoU represents the intersection-to-union ratio. The Wise loU loss can be defined as follows:

2 $${L}_{WIoU}={R}_{WIoU}\times {L}_{IoU}$$

3 $${R}_{WIoU}=exp\left(\frac{{\left(x-{x}_{gt}\right)}^{2}+{\left(y-{y}_{gt}\right)}^{2}}{{\left({W}_{g}^{2}+{H}_{g}^{2}\right)}^{*}}\right)$$

Among them, ${R}_{WIoU}\in \left[1\right.\left.,e\right)$ significantly amplifies the ${L}_{IoU}$ of anchor boxes with ordinary quality. However, ${L}_{IoU}\in \left[\mathrm{0,1}\right]$ significantly reduces ${R}_{WIoU}$ for high-quality anchor boxes. This adjustment reduces the emphasis on center point distance when the anchor box overlaps well with the target box. To prevent ${R}_{WIoU}$ from the generation of gradients that obstruct the attainment of convergence, the calculation separates ${W}_{g}$ and ${H}_{g}$, effectively eliminating factors that hinder convergence during training.

The definition of HIoU proposed in this study is as follows:

4 $${L}_{HIoU}={R}_{WIoU}\times {L}_{IoU}+\frac{1}{2}\left(\frac{{\left(x-{x}_{gt}\right)}^{2}+{\left(y-{y}_{gt}\right)}^{2}}{{W}_{g}^{2}+{H}_{g}^{2}}+\alpha \gamma \right)$$

5 $$\alpha =\frac{\gamma }{\left(1-IoU\right)+\gamma }$$

6 $$\gamma =\frac{4}{{\pi }^{2}}{\left(arctan\frac{{w}_{gt}}{{h}_{gt}}-arctan\frac{w}{h}\right)}^{2}$$

Compared to CIoU and Wise-loU, the HIoU proposed in this study offers the advantage of dynamically adjusting the loss for bounding box regression, similar to Wise-loU. During the early stages of training, when the IoU between the predicted candidate box and the real object annotation box is relatively low, the model should prioritize improving this IoU. To achieve this, the first term ${R}_{WIoU}$ in the aforementioned ${R}_{WIoU}\times {L}_{IoU}$ effectively amplifies the model’s penalty for IoU. As the training progresses and the IoU between the predicted candidate box and the real object annotation box reaches a high degree of overlap, further significant changes are minimal. Consequently, in this later stage, the first term ${L}_{IoU}$ in the IoU formulation ${R}_{WIoU}\times {L}_{IoU}$ is deemphasized. As a result, the value of IoU decreases, allowing the model to automatically focus on regressing the candidate box towards the center point and adjusting the aspect ratio. This automatic adjustment facilitates the regression of the predicted candidate box towards the real object annotation box, ultimately enhancing the model’s performance.

HIoU incorporates improvements in the intersection and union operations of traditional IoU by dynamically adjusting the regression loss of bounding boxes. By doing so, it mitigates the penalties for geometric metrics like distance and aspect ratio. This approach enables a more comprehensive consideration of multiple factors including IoU, position, size, shape, etc., relating to the anticipated box and the actual box. Consequently, it enhances the precision of object detection. In the HIoU formulation, interventions occur at a lower level by reducing the weight of the second and third terms in the loss function, which corresponds to penalizing geometric measurements. Although this intervention increases during model training, it improves the generalizing capacity of the model, allowing it to perform better in varying scenarios.

In summary, HIoU demonstrates improved adaptability and robustness in specific scenarios compared to CIoU and Wise-loU. It offers enhanced evaluation capabilities for object detection tasks. By dynamically adjusting the regression loss and considering multiple factors, HIoU provides a more comprehensive and accurate assessment of performance in these tasks. Its advantages contribute to its effectiveness in evaluating and enhancing object detection performance.

## Results

### Experimental environment

This research employed Python programming language and leveraged GPU acceleration to conduct the experiments. Details of the experimental environment are provided in Table 2.

**Table 2:** Experimental environment.

No	Category	Model
1	Operating system	Windows 10
2	Development environment	CUDA 11.4.153
3	Graphics card (GPU)	NVIDIA GeForce GTX 3090 Ti, 24 GB VRAM

### Evaluation indicators

The main evaluation metrics for object detection algorithms include detection accuracy, model complexity, and detection speed.

Detection accuracy is assessed primarily through precision (P), recall (R), and mean average precision (mAP). The subsequent equations can be utilized to compute these metrics.

7 $$P=\frac{TP}{TP+FP}$$

8 $$R=\frac{TP}{TP+FN}$$

9 $$mAP=\frac{\sum_{i=1}^{k}{AP}_{i}}{K}$$

In the aforementioned formulas, the meanings of each variable are as follows:

True Positive (TP): Signifies the algorithm's accurate detection of the actual number of existing disease targets. In other words, the algorithm successfully and precisely labels real disease targets as such.

False Positive (FP): Represents the algorithm's erroneous labeling of non-existent disease targets as disease instances. In essence, the algorithm incorrectly identifies non-disease areas as disease-infected.

False Negative (FN): Indicates the algorithm's failure to accurately detect the actual number of existing disease targets. Specifically, the algorithm falls short in correctly identifying real disease targets as disease instances.

K: Denotes the total number of categories. The mAP (mean average precision) is the average value of precision for all categories, where the precision for each category is represented by its corresponding AP (average precision).

The complexity of an object detection algorithm is indicated by the volume of the model, the number of parameters, and the computational complexity. A higher value in these aspects signifies higher model complexity. In this study, the evaluation indicators for model complexity include model computation and model size. Model computation represents the time complexity and is measured using Floating-Point Operations (FLOPs). A higher computational complexity indicates a greater requirement for computational resources.

Model size reflects the spatial complexity. It measures the amount of storage space needed to store the model parameters. By considering both computational and spatial complexities, we can gauge the resource requirements and efficiency of an object detection algorithm.

Frame per second (FPS) is an essential evaluation metric for measuring the detection speed of an algorithm. A higher FPS value indicates faster processing speed, enabling real-time or near-real-time object detection in video or image streams. Evaluating FPS helps assess the efficiency and effectiveness of an object detection algorithm in handling real-time applications where prompt and accurate detection is crucial.

### Model training

When training the network model for vegetable disease object detection, the dimensions of the input image are uniformly modified to 640 × 640 × 3. To improve training efficiency and accelerate convergence, the model employs a freezing training method. The basic parameter settings used are provided in Table 3. The SGD optimizer is utilized with a total of 300 epochs. During the initial 50 epochs, the backbone parameters are kept fixed and not updated. For the remaining 250 epochs of thawing training, the parameters are reduced. Furthermore, to enhance the detection capability, Mosaic and Mixup data augmentation techniques are turned off during the last 100 training epochs. This adjustment aims to refine the performance of the model in detecting vegetable diseases.

**Table 3:** Training parameter settings.

Training stage	Epoch	Batch size	Learning rate	Optimizer
Freezing phase	50	32	0.001	SGD
Thawing phase	250	16	0.0001	SGD

**Figure 11:**
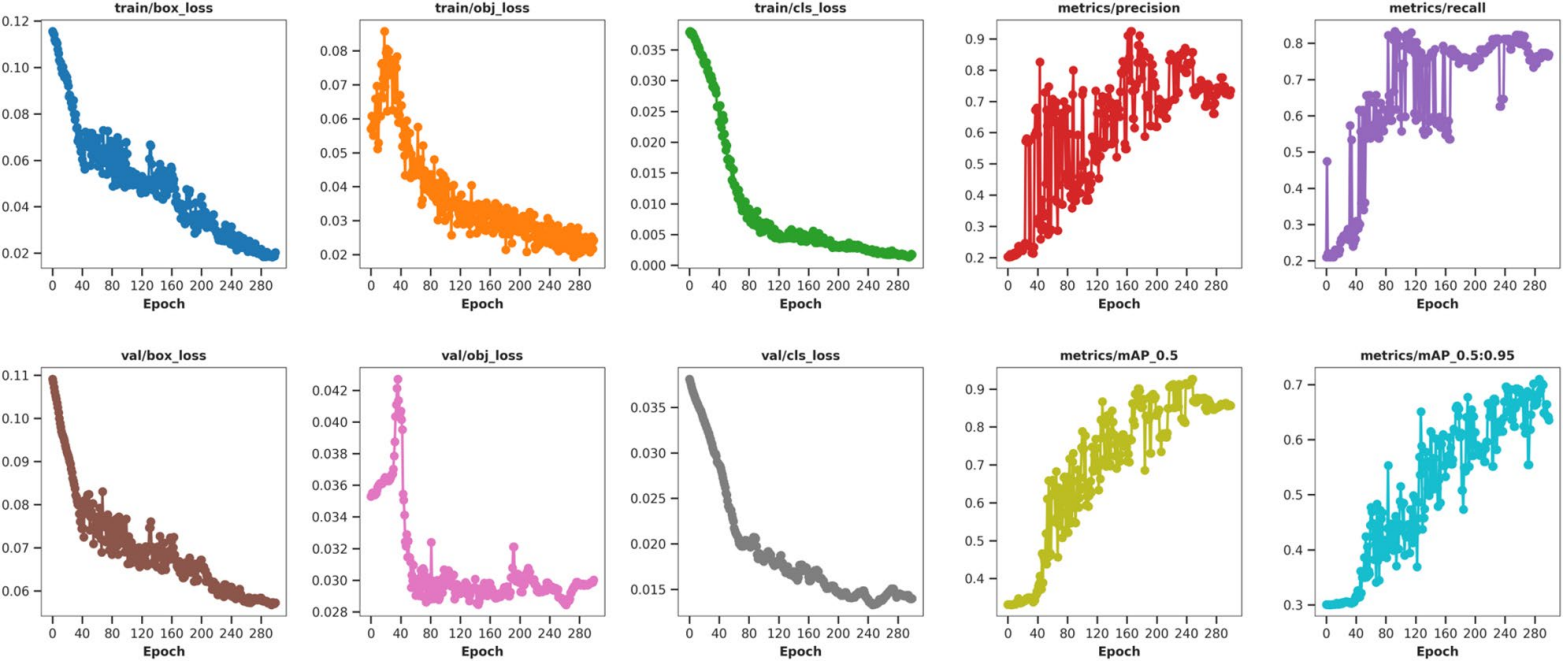
Training results of the proposed YOLOv8n-vegetable.

As shown in Fig. 11, during the training process, the values of the loss functions exhibit a decreasing trend, indicating that the SGD optimizer continuously optimizes the model by updating the network weights and other parameters. Prior to 80 epochs, there is a rapid decrease in the values of the loss functions, accompanied by a swift improvement in precision, recall, mAP@0.5, and mAP@0.5:0.95. Around 120 epochs, the rate of decrease in the loss function values gradually slows down. Similarly, the improvements in precision, recall, mAP@0.5, and mAP@0.5:0.95 also show a deceleration. Upon reaching 250 epochs, the training loss curve exhibits almost no further decrease, and other metric values tend to stabilize, indicating that the network model has essentially converged. At the conclusion of training, the optimal network weights are obtained, thus demonstrating the effectiveness of the model.

### Comparative experiments on multiple mainstream and lightweight object detection models

To demonstrate the superiority of the proposed YOLOv8n-vegetable algorithm over existing popular object detection models, we conducted comparative experiments. We selected both mainstream and lightweight object detection models, and introduced modifications to the YOLOv8 algorithm by incorporating lightweight backbone networks. The comparison results are presented in Table 4. Evaluation criteria included Precision, Recall, mAP, Parameters, and Model size, with mAP assessed at an IoU (Intersection over Union) value of 0.5. All comparisons were conducted under identical conditions, utilizing unified configurations and the same dataset. The performance of these models was evaluated alongside YOLOv8n-vegetable, considering the aforementioned metrics. This comparison aims to determine the superior capabilities of YOLOv8n-vegetable in vegetable disease object detection, providing insights into its performance relative to other widely adopted models.

**Table 4:** Comparative experiments on multiple mainstream and lightweight object detection models.

Algorithm	Precision/%	Recall/%	mAP (%)	Parameters/G	Model size/MB	FPS
Faster R-CNN	87.44	81.39	84.46	30.46	112.71	97.10
YOLOv3-tiny	85.32	79.88	83.29	10.85	20.71	117.01
YOLOv4-tiny	87.80	82.13	85.37	11.23	28.00	126.04
YOLOXs	89.29	81.59	87.40	11.06	38.24	144.07
YOLOv5s	92.97	86.23	91.70	9.22	17.77	174.05
YOLOv6	92.96	86.29	91.75	9.14	17.76	174.02
Yolov7-tiny	67.84	63.44	58.07	8.16	15.57	134.07
Yolov8n	83.24	83.22	86.45	5.05	9.28	275.05
YOLOv8n-FastNet	76.11	72.96	74.14	4.25	8.66	283.32
YOLOv8n -MobileNet	75.98	73.87	75.22	4.69	8.92	276.13
YOLOv8n-vegetable	92.72	87.73	92.91	4.89	9.07	271.07

Based on the findings presented in Table 4, the original YOLOv8n algorithm exhibits superior precision, recall, and mAP compared to Faster R-CNN, YOLOv3-tiny, YOLOv4-tiny, YOLOXs, YOLOv6, and YOLOv7-tiny. In particular, YOLOv8n achieves these results while having a smaller parameter quantity and model size than the other six networks. Although YOLOv5s slightly outperforms YOLOv8n in terms of accuracy, it comes at the cost of nearly double the parameter quantity and model size compared to YOLOv8n.

YOLOv8n-FastNet and YOLOv8n-MobileNet are improved versions of the YOLOv8n network, with the backbone networks replaced by FastNet35^38^ and MobileNet^39^, respectively. It is evident that both exhibit a significant reduction in parameters and model size. Despite the clear advantages in lightweight design, they result in respective performance decreases of 12.31% and 11.23% in mAP. Thus, it is apparent that solely modifying the backbone network is insufficient, necessitating further optimization. To meet the requirements of vegetable disease detection in greenhouse planting environments, the proposed model must effectively balance lightweight design and performance while surpassing some common algorithms.

In contrast, the proposed YOLOv8n-vegetable algorithm, which is based on the original YOLOv8n, maintains a similar FPS with even smaller parameter quantities and model sizes. Specifically, it reduces the parameter quantity by 0.16G and the model size by 0.21 MB compared to the original model. Additionally, the precision of the YOLOv8n-vegetable exceeds that of the original YOLOv8n algorithm, with an increase of 6.46% in average accuracy (mAP). These improvements in various indicators demonstrate the superiority of the proposed algorithm.

Through comparative experiments, it is evident that the proposed YOLOv8n-vegetable performs exceptionally well. It achieves a reduction in both parameter count and model size while maintaining a high mAP, making it the most efficient among the compared models. The FPS also experiences a significant improvement, meeting the speed requirements for detection tasks. This accomplishes lightweight objectives under high precision conditions. The model's enhancements include the introduction of GhostConv from the GhostNet network and the novel C2fGhost module, reducing computational and parameter loads. The integration of the Occlusion Perception Attention Module (OAM) ensures effective detection of occluded disease targets. The addition of a small target detection layer improves detection capability and accuracy for smaller targets. The introduction of the HIoU loss function contributes to enhanced speed and accuracy. Through various comparative experiments, this model emerges as a high-precision, fast, and lightweight solution, showcasing promising applications.

### Comparative experiment on the detection effect of different categories of vegetable diseases

To evaluate the capability of the model to extract and distinguish features related to different categories of vegetable diseases, we compared the detection results (average precision − AP values) of YOLOv8n and the proposed model for each category. The comparison is presented in Table 5.

**Table 5:** Detection precision for different vegetable disease categories.

No	Vegetable type	Disease category	AP (%)	
			YOLOv8n	YOLOv8n-vegetable
A1	Tomato	Healthy	95.87	98.98
A2	Tomato	Early blight	89.76	92.07
A3	Tomato	Late blight	85.18	93.01
A4	Tomato	Gray mold	81.53	91.06
A5	Tomato	Leaf mildew	83.11	91.01
A6	Tomato	Leaf spot	82.81	90.02
A7	Tomato	Ulcer disease	84.22	90.08
A8	Tomato	Anthracnose	79.58	86.06
A9	Tomato	Leaf curl	82.03	88.09
A10	Tomato	Viral disease	84.43	87.02
B1	Cucumber	Healthy	95.26	98.66
B2	Cucumber	Powdery mildew	83.01	90.02
B3	Cucumber	Downy mildew	81.49	90.10
B4	Cucumber	Brown spot	79.88	86.03
B5	Cucumber	Anthracnose	82.92	88.01
B6	Cucumber	Viral disease	84.70	86.00
C1	Eggplant	Healthy	95.38	98.87
C2	Eggplant	Verticillium wilt	85.64	91.05
C3	Eggplant	Brown spot	81.38	85.06
C4	Eggplant	Viral disease	85.07	89.04

Referring to the results outlined in Table 5, the YOLOv8n-vegetable shows improved detection accuracy for 20 types of vegetable disease objects compared to the original YOLOv8n model. In particular, significant improvements are observed for small disease objects, such as tomato late blight, tomato gray mold, tomato leaf mold, tomato leaf spot, cucumber powdery mildew and cucumber downy mildew. The average precision for these disease objects is respectively 7.83%, 9.53%, 7.90%, 7.22%, 7.00%, and 8.61% higher than that of YOLOv8n. Furthermore, the proposed model demonstrates substantial accuracy improvements for disease objects that undergo notable changes in size and shape, such as cucumber anthracnose and eggplant brown spot. These findings suggest that the network design of YOLOv8n-vegetable model is well suited for extracting crucial feature information from the feature layer, thus enhancing the accuracy of object detection.

### Ablation study on each improved module

To assess the efficacy of the improvement strategies of the proposed YOLOv8n-vegetable, ablation experiments were conducted using the original YOLOv8n model as the baseline. The evaluation indicators used in these experiments included Precision, Recall, mAP, Parameters, and Model size. Different combinations of multiple improvement modules were tested, and the outcomes are presented in the table below.

Based on the findings presented in Table 6, several improvement modules were added to the YOLOv8n-vegetable model, resulting in significant improvements in various indicators:

1. After adding the lightweight GhostConv and the designed C2fGhost, the mAP increased by 4.16%. Additionally, there was a reduction in model parameters by 0.37G and model size by 1.17 MB.
2. The incorporation of the OAM attention mechanism resulted in a 0.6% improvement in precision, 1.27% in recall, and 1.03% in mAP. This showcases that the proposed OAM enhances feature maps, allowing the network to concentrate more on extracting features from visible regions of vegetable diseases in the input image. Consequently, this diminishes occlusion impact on the model's inference process, thereby enhancing robustness to occlusion in the greenhouse planting environment.
3. Although the GAM attention mechanism resulted in a decrease in some indicators compared to the addition of OAM, the decision was made to choose OAM as it yielded a better overall performance.
4. Incorporating a layer specifically designed for detecting small-sized objects resulted in a 1.93% rise in Precision, a 1.73% surge in Recall, and a 1.42% increase in mAP.s
5. Integrating the HIoU loss function further improved precision, recall, and mAP by 0.1%, 1.66% and 0.88%, respectively.

**Table 6:** Ablation experiment on each improved module.

GhostNet	OAM	GAM	SDL	HIoU	Precision/%	Recall/%	mAP (%)	Parameters/G	Model size/MB	FPS
No	No	No	No	No	83.24	83.22	86.45	5.05	9.28	275.05
Yes	No	No	No	No	90.69	84.34	90.61	4.68	8.01	216.06
Yes	No	Yes	No	No	90.82	84.89	91.25	4.72	8.32	187.02
Yes	Yes	No	No	No	91.29	85.61	91.64	4.79	8.36	195.08
Yes	Yes	No	Yes	No	92.62	86.07	92.03	4.83	8.98	245.08
Yes	Yes	No	Yes	Yes	92.72	87.73	92.91	4.89	9.07	271.07

The experimental findings provide evidence that the improved YOLOv8n-vegetable, with the inclusion of these proposed improvement modules, achieved significant advances across all evaluation indicators in comparison to the original YOLOv8n model. This verifies the efficacy of the improvement modules.

### Ablation study on attention mechanisms

In this section, we conduct ablative experiments on different attention mechanisms embedded in the network model to further validate the effectiveness of the proposed occlusion perception attention module. We choose YOLOv8n as the baseline model and conducted ablation experiments by adding SE, CBAM, GAM, Biformer, and the proposed OAM. We compare the increase in parameters and computational cost, as well as the final detection accuracy, resulting from different attention mechanisms. The results are presented in Table 7.

**Table 7:** Ablation experiment on attention mechanisms.

YOLOv8n	Parameters/G	FLOPs (B)	mAP (%)
+ SE	+ 0.009	7.1	88.68
+ CBAM	+ 0.013	7.3	89.07
+ GAM	+ 1.791	10.7	89.39
+ Biformer	+ 0.265	19.2	87.32
+ OAM	+ 0.001	7.2	90.01

It can be observed that SE and CBAM achieved a slight improvement in accuracy with a small increase in parameters and computational cost. GAM exhibited a significant improvement in accuracy, but at the expense of a larger parameter count. On the other hand, Biformer introduced a substantial increase in computational cost while leading to a decrease in accuracy. In contrast, OAM showed a negligible increase in computational cost and resulted in a significant improvement in accuracy with minimal increase in parameters.

### Ablation study on loss function

**Figure 12:**
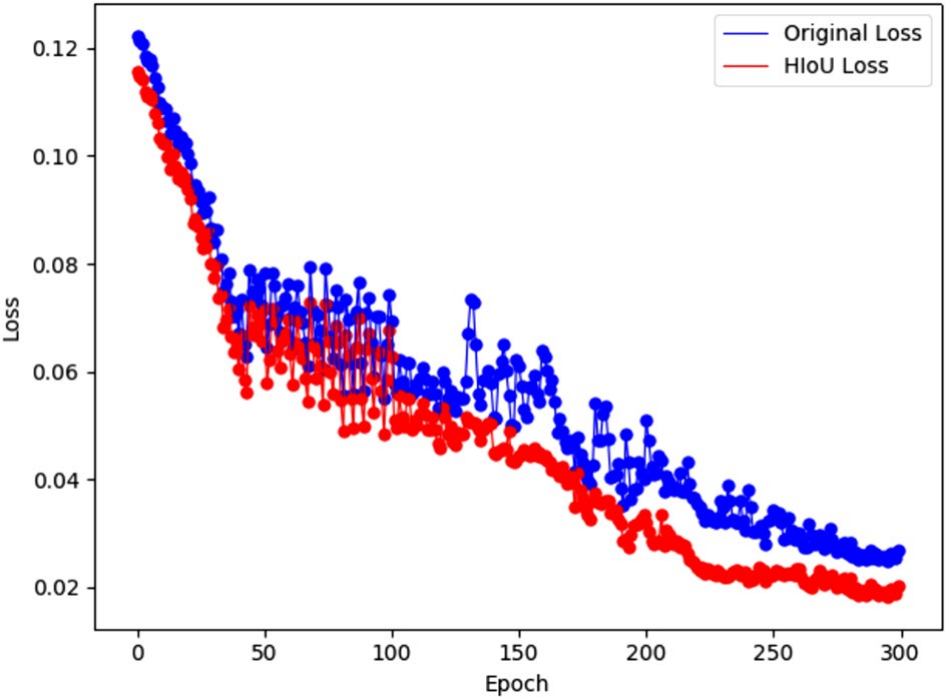
Comparison of training loss curves.

To validate the effectiveness of the optimized loss function, YOLOv8n is compared with the experimental results of only improving the loss function. To more intuitively demonstrate the enhancement of model convergence ability due to the optimization of the loss function, the regression loss during the training process is visualized, as shown in Fig. 12. It can be seen that the optimized loss function converges faster.

### Results of the YOLOv8n-vegetable model on uncropped images

Since the images in the self-built dataset have been cropped, to evaluate the YOLOv8n-vegetable model's performance on the unedited images, this study tests the model on the full image. The results are presented in Table 8.

**Table 8:** Statistical results of the proposed YOLOv8n-vegetable on uncropped images.

No	Vegetable type	Disease category	AP (%)
A1	Tomato	Healthy	97.01
A2	Tomato	Early blight	88.86
A3	Tomato	Late blight	88.60
A4	Tomato	Gray mold	88.87
A5	Tomato	Leaf mildew	89.64
A6	Tomato	Leaf spot	88.89
A7	Tomato	Ulcer disease	88.80
A8	Tomato	Anthracnose	85.78
A9	Tomato	Leaf curl	87.61
A10	Tomato	Viral disease	84.25
B1	Cucumber	Healthy	94.38
B2	Cucumber	Powdery mildew	88.08
B3	Cucumber	Downy mildew	86.79
B4	Cucumber	Brown spot	84.53
B5	Cucumber	Anthracnose	87.90
B6	Cucumber	Viral disease	82.60
C1	Eggplant	Healthy	97.91
C2	Eggplant	Verticillium wilt	90.07
C3	Eggplant	Brown spot	83.80
C4	Eggplant	Viral disease	86.07

From Table 8, the accuracy of the YOLOv8n-vegetable model running on the unedited images is high for different categories of vegetable diseases, all exceeding 82%. The experimental results indicate that the proposed method achieves good performance on actual greenhouse vegetable disease image data. It can provide technical assistance and support for the intelligent management and prevention of vegetable diseases in the greenhouse planting environment.

#### Visual representation of detection outcomes

**Figure 13:**
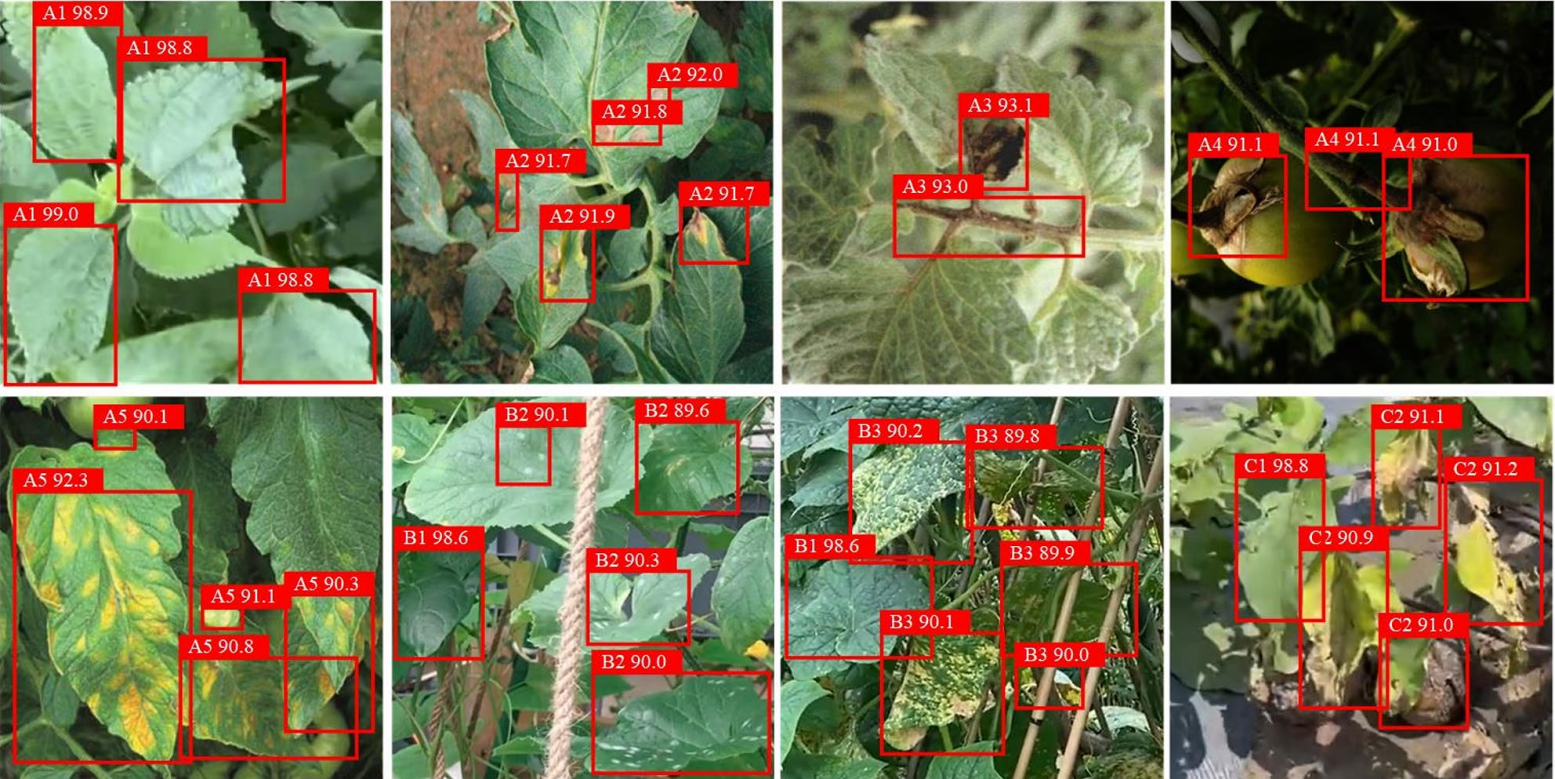
Visualization of detection results.

To clearly demonstrate the efficacy of the YOLOv8n-vegetable model, a set of different images of vegetable diseases was used as input to showcase the algorithm’s capability in accurately extracting vegetable disease features within complex greenhouse planting environments. The detection results are visually presented in Fig. 13.

In Fig. 13, the original vegetable disease images are shown, with bounding boxes outlined around the identified regions of the disease. Each bounding box is labeled with the corresponding disease class for easy identification. The accurate localization and identification of vegetable diseases in the images displayed demonstrate the effectiveness of the YOLOv8n-vegetable model in detecting various types of diseases in challenging agricultural settings.

The findings illustrate that the YOLOv8n-vegetable shows exceptional superiority in detecting diseases across various scales, particularly excelling in the detection of small-scale diseases without any instances of missed detection. Consequently, the proposed algorithm is highly suitable for vegetable disease detection tasks that involve scale variations. Furthermore, the enhancements in the YOLOv8n-vegetable model in handling occluded objects enable it to effectively extract features from occluded vegetable diseases. As a result, the proposed YOLOv8n-vegetable model satisfies the detection requirements for vegetable diseases within complex greenhouse planting environments. These findings highlight the robustness and adaptability of the YOLOv8n-vegetable model, demonstrating its potential for real-world application in the field of vegetable disease detection and management.

## Discussion

Vegetable disease detection plays a crucial role in intelligent crop protection. In this study, we present an efficient and lightweight YOLOv8n-vegetable model designed specifically for the detection of vegetable diseases. To decrease the size of the model, we incorporate the GhostConv module and the redesigned C2fGhost module. Furthermore, an occlusion perception attention module is incorporated to improve the model’s capability to enhance feature fusion and feature extraction. Additionally, a small-sized object detection layer is introduced to boost accuracy in detecting small-sized objects. By utilizing the HIoU loss function, the proposed model demonstrates improved performance in bounding box regression.

The experimental results of the proposed YOLOv8n-vegetable model on a self-built dataset for vegetable disease detection indicate advantages compared to some current mainstream object detection and lightweight methods. It excels in evaluation metrics such as Precision, Recall, mAP, Parameters, Model size, and FPS. The proposed approach achieves an mAP of 92.91% and a speed of 271.07 frames per second, demonstrating its competence in vegetable disease detection tasks within greenhouse planting environments.

## Conclusion

The YOLOv8n-vegetable model proposed in this study employs an end-to-end prediction approach, which has several advantages such as high detection accuracy, fast processing speed, and easy deployment. It effectively generates marker boxes and corresponding disease category labels for the detected regions, facilitating the automated prevention and control of vegetable diseases. As a result, the workload of disease detection and control for farmers is significantly reduced. Subsequent work will concentrate on streamlining the proposed model and developing embedded hardware platforms. This will enable the model to perform video capture and intelligent detection functions locally, leading to faster disease alerts. The ultimate objective is to establish a control linkage with agricultural IoT devices, allowing for timely disease alerts when they occur.

## Data Availability

The data utilized in this paper is obtained through self-gathering and is made publicly available (a part of it) to make the study reproducible. It can be accessed at https://github.com/tyuiouio/plant-disease-detection-in-real-field. If you want to request the complete dataset and code, please email the corresponding author.
